# CT and MRI findings of intra-parenchymal and intra-ventricular schwannoma: a series of seven cases

**DOI:** 10.1186/s12880-022-00917-z

**Published:** 2022-11-17

**Authors:** Zhen-yi Zhang, Zhi-qing Mo, You-ming Zhang, Hong Yang, Bin Yao, Hao Ding

**Affiliations:** 1 Department of Radiology, Xiangya Hospital, Central South University, 87 Xiangya Road, Changsha, 410008 People’s Republic of China; 2 Department of Radiology, Guilin People’s Hospital, Guilin, Guangxi 541000 People’s Republic of China; 3 Department of Radiology, Nanxishan Hospital of Guangxi Zhuang Autonomous Region, No. 46 Chongxin Road, Xiangshan District, Guilin City, Guangxi Zhuang Autonomous Region (GZAR) People’s Republic of China; 4 Department of Radiology, The Second Affiliated Hospital of Nanchang University, No.1 Minde Road, Nanchang, 330006 Jiangxi China; 5 Department of Radiology, Affiliated Hospital of Guilin Medical University, No.15 Lequn Road, Xiufeng District, Guilin City, Guangxi 541001 People’s Republic of China

**Keywords:** Intracranial schwannoma, Intra-ventricular schwannoma, Imaging feature, Magnetic resonance imaging, Computed tomography

## Abstract

**Objective:**

To analyze the computed tomography (CT) and magnetic resonance imaging (MRI) features of patients with intra-parenchymal and intra-ventricular schwannoma.

**Methods:**

The CT and MRI features of seven cases with intra-parenchymal and intra-ventricular schwannoma were analyzed retrospectively.

**Results:**

There were four men and three women (median age, 25 years; range, 12–42 years) in this study. The median tumor size was 4.4 cm (range, 3.1–6.5 cm). The mass was, respectively, round in four cases (57.1%), lobulated in two cases (28.6%) and oval in one case (14.3%). All tumors were well-circumscribed. Septa in the mass could be observed in three cases (42.9%), and nodular calcification was observed in two cases (28.6%), which peritumoral edema (n = 3, 42.9%) and hydrocephalus (n = 3, 42.9%) could be observed. Most of these lesions (n = 6) presented iso-hypointensity on T1-weighted images and iso-hyperintensity on T2-weighted images, except one lesion showing low intensity on T2WI. In addition, a fluid–fluid level was observed in one case. After contrast agents’ injection, all masses illustrated heterogeneously moderate to marked enhancement.

**Conclusions:**

A well-defined solid and cystic mass with calcification and moderate to marked delayed enhancement may be an objective account of intra-parenchymal or intra-ventricular schwannoma.

**Supplementary Information:**

The online version contains supplementary material available at 10.1186/s12880-022-00917-z.

## Introduction

Intracranial schwannomas, closely associated with the acoustic nerve, are usually located at the cerebellopontine angle. Unrelated to cranial nerves, such as intraparenchymal and intraventricular entities, it is extremely rare for our routine clinical practice[1]. The first case of the English language medical literature was reported by Gibson in 1966 [2]. Several cases have been reported since then. However, there are few studies to describe the imaging characteristics of such tumors in detail [3–5].

The preoperative imaging diagnosis of a brain tumor is crucial for the surgical plan. If the lesion on magnetic resonance imaging (MRI) is suggestive of malignancy, extended brain parenchymal resection around the tumor is required. On the contrary, if the lesion is diagnosed as a benign entity, aggressive surgical treatment would be unnecessary. Therefore, correct preoperative diagnosis of intraparenchymal and intraventricular schwannoma is crucial to avoid unnecessary extended surgical treatment. However, due to the insufficient knowledge about its radiological features, it is still easy to misdiagnose intraparenchymal and intraventricular schwannomas as other malignant tumors; As a result, it is of vital significance for surgeons to investigate the imaging features of intraparenchymal and intraventricular schwannoma.

In this study, we retrospectively collected the clinical and radiological data of a series of patients with intracranial schwannomas who underwent MRI and/or computed tomography (CT) examinations, to analyze the characteristic imaging features in detail. We estimate this retrospective analysis of the intraparenchymal and intraventricular schwannoma would provide the surgeon with useful preoperative information.

## Materials and methods

### Patients

This retrospective study was approved by the institutional review board of our hospital, with the requirement for informed consent waived. From January 2010 to November 2019, intra-parenchymal and intra-ventricular schwannoma at our hospital were surgically and pathologically proved based on the clinical trials of seven patients. One patient underwent CT examination, one patient had undergone MRI and CT, while the remaining five underwent MRI. Clinical and radiologic data were collected for further analysis.

### Imaging techniques

The head CT examination was performed with a 320-detector volume CT system (Aquilion ONE, Toshiba, Otawara, Japan). MRIs were obtained with a 1.5-T and 3.0-Tsuperconductive unit (Singa HDxt [GE Medical Systems, Umatilla, Florida, USA]), and 8 channel head coil was adopted. The sequences included sagittal T2-weighted images (T2WI), axial T2-FLAIR and transverse T1/T2-weighted images. Contrast-enhanced sagittal, coronal, and transverse T1-weighted enhanced (T1WI) images were obtained after an intravenous injection of gadopentetate dimeglumine (Magnevist [Schering, Berlin, Germany]) at a dose of 0.2 mmol/kg and rate of 1.5 mL/second.

### Imaging data analysis

The images were analyzed by two radiologists with respective 4 and 5 years of experience in the brain imaging. CT or MR characteristics of the imaging data, including location, shape and size, margin, attenuation, and enhancement pattern were independently evaluated. Any discrepancy was resolved by consensus.

## Results

### Clinical data

The study group was formed by four men and three women with a median age of 25 years (range, 12–42 years).Clinical symptoms included headache (2 cases), dizziness (1 case), dizziness and headache (1 case), diplopia dizziness and headache (1 case), epilepsy dizziness and vomiting (1 case), incidental findings (1 case).

### Imaging findings

The imaging findings of the seven cases with intra-parenchymal and intra-ventricular schwannoma are shown in Table 1.

**Table 1:** CT and MRI Imaging Features of 7 cases with intra-parenchymal and intra-ventricular schwannoma

case	site	size (cm^2^)	gender/age (years)	modality	texture	margin	cal	density	T1WI	T2WI	cystic degeneration	septa	hydrocephalus	delayed progressive enhancement
1	frontal lobe/R	6.1 × 6.5	M/12	MRI	cystic and solid	clear	–	–	iso-hypo	hypo/hyper	+	+	+	+
2	fourth ventricle	4.2 × 3.6	F/24	MRI	cystic and solid	clear	–	–	hypo	hyper	+	–	–	+
3	pineal gland	5.9 × 5.2	F/33	CT and MRI	cystic and solid	clear	+	iso-hypo	hypo	hyper	+	–	+	+
4	Cerebellum /L	4.3 × 3.5	M/42	CT	cystic and solid	clear	+	iso-hypo	hypo	hyper	+	+	−	+
5	Lateral ventricle/L	3.1 × 3.1	M/14	MRI	cystic and solid	clear	–	–	hypo	hyper	+	+	+	+
6	cerebral peduncle/L	3.2 × 3.1	M/37	MRI	cystic and solid	clear	–	–	hypo	hyper	+	–	–	+
7	frontal lobe/R	4.2 × 4.6	F/16	MRI	cystic and solid	clear	–	–	hypo	hyper	+	–	–	+

R: right; L: left; M:male; F:female; cal: calcification; iso: isodensity/isointensity; hypo: hypodensity/hypointensity; hyper:hyperintensity; '-' denotes negative; ‘ + ’ denotes positive

**Fig. 1:**
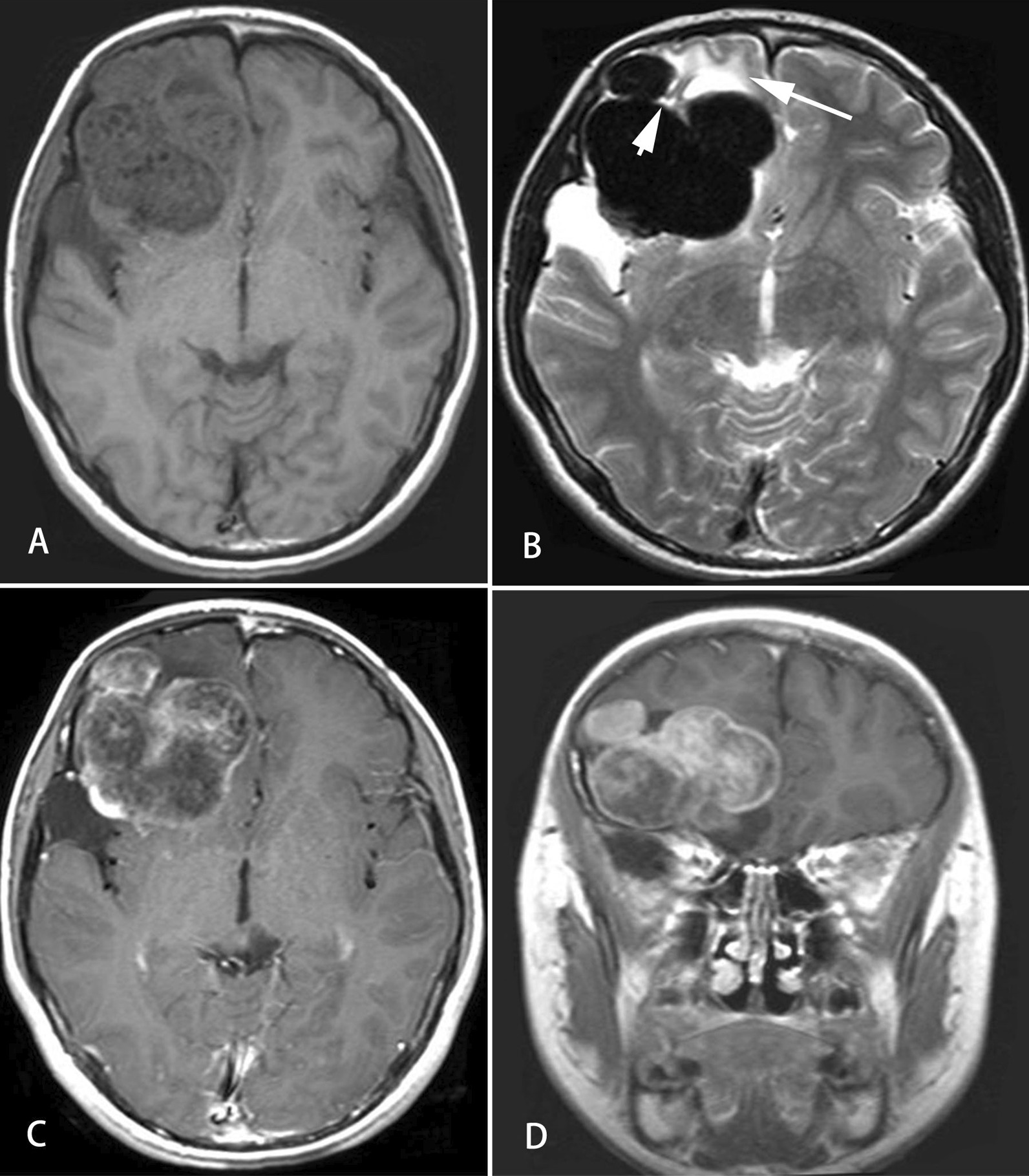
MRI findings of a 12-year-old man with intraparenchymal schwannoma. A well-defined heterogeneous mass with peritumoral edema (arrow) and cystic degeneration (arrow head) is observed in the right frontal lobe (**A** and **B**). The mass shows hypo-intensity on T1 WI and hypo-/hyper-intensity on T2WI. The hypo-intensity area on T1WI and T2WI was suggestive of calcification (**B**). Heterogeneous, marked and delayed enhancement could be observed on the axial and coronal T1WI (**C** and **D**)

**Fig. 2:**
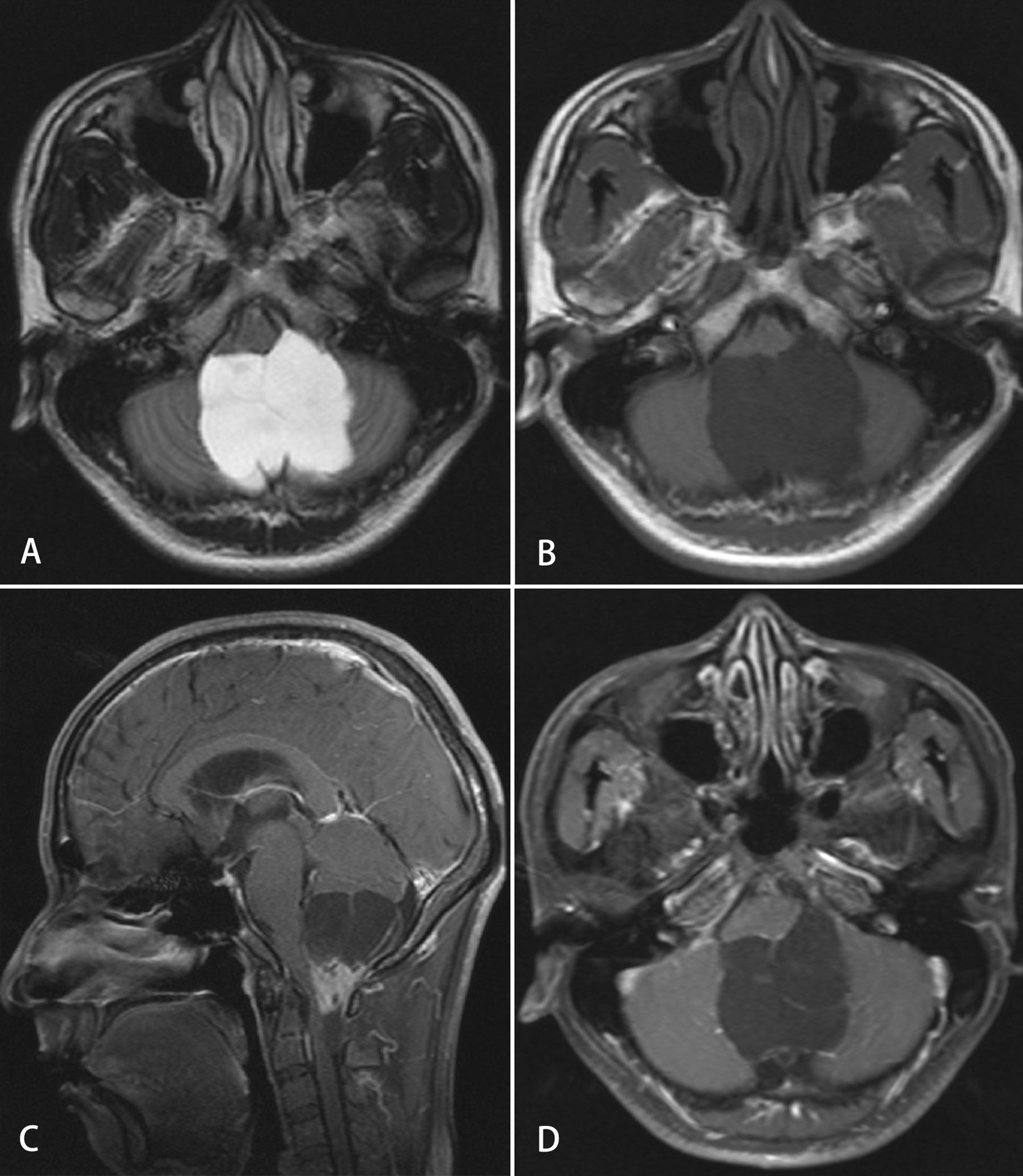
MRI findings of a 27-year-old man with intraparenchymal schwannoma. A well-defined solid and cystic mass with septa could be observed in the vermin of the cerebellum (**A**, **B**). The solid components and the septa are enhanced after the contrast agents’ injection (**C**, **D**)

**Fig. 3:**
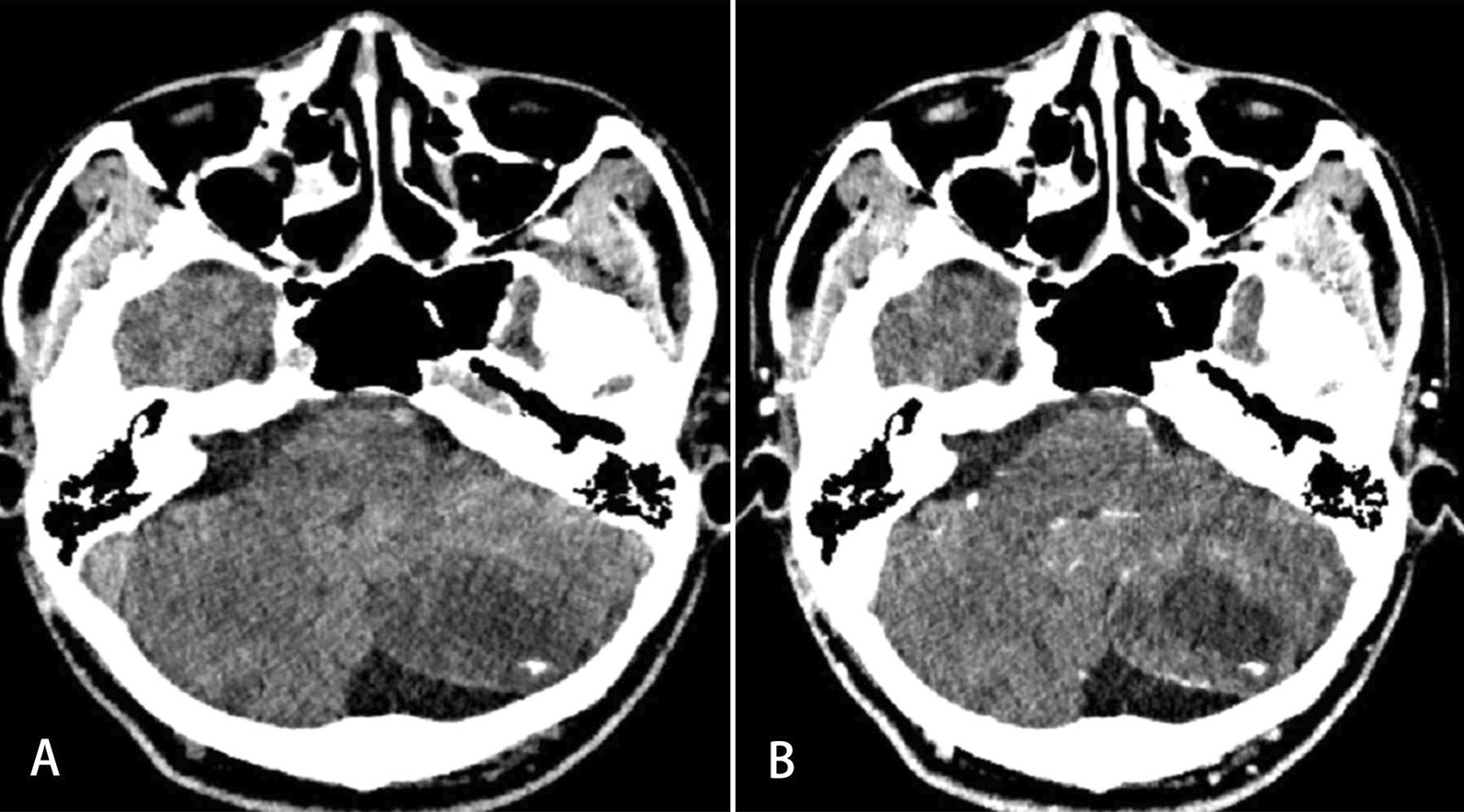
CT findings of a 42-year-old man with intraparenchymal schwannoma. Nodular calcification could be observed along the edge of the solid and cystic mass in the left cerebellar hemisphere (**A**, **B**)

In our study, CT findings were available for two cases while MRI was performed in six other cases. The median tumor size was 4.4 cm (range, 3.1–6.5 cm) in our patients. The mass shape was round in four cases (57.1%), lobulated in two cases (28.6%) and oval in only one case (14.3%). All tumors were presented with well-circumscribed margins (Fig. 1). Septa (Fig. 2) could be observed in three cases (42.9%). Nodular calcification (Fig. 3) could be located in two cases (28.6%) by CT examination. Peritumoral edema exists in three patients (42.9%). Hydrocephalus was presented at three patients (42.9%). The CT attenuation and MR signal intensity of tumors in all our patients were heterogeneous. Most of these lesions demonstrated iso-hypointensity on T1WI and iso-hyperintensity on T2WI. Yet solid component of the lesion in Case 1 showed low intensity on T2WI. Besides, a fluid–fluid level was visible in the lesion of Case 7.

After administration of contrast agent, the enhancement of all masses was heterogeneously delayed moderate or marked enhancement (Fig. 1). Most of these cases indicated solid component enhancement. Besides, cystic wall and solid component enhancement could be observed in Case 2.

### Surgical and pathological findings

At surgery, five tumors were intra-parenchymal (including two cased in the right frontal lobes, one case in pineal gland, one case in the left cerebellar hemisphere, and one case in the left cerebral peduncle) and the remaining two lesions were intra-ventricular (including one case in the fourth ventricle and the other case in the left lateral ventricle). The seven masses were rounded or lobulated, firm, well-defined, with a tan-yellow to greyish-white appearance. All tumors, especially the larger mass, were heterogeneous in texture, with cystic degeneration, calcification, and septa.

**Fig. 4:**
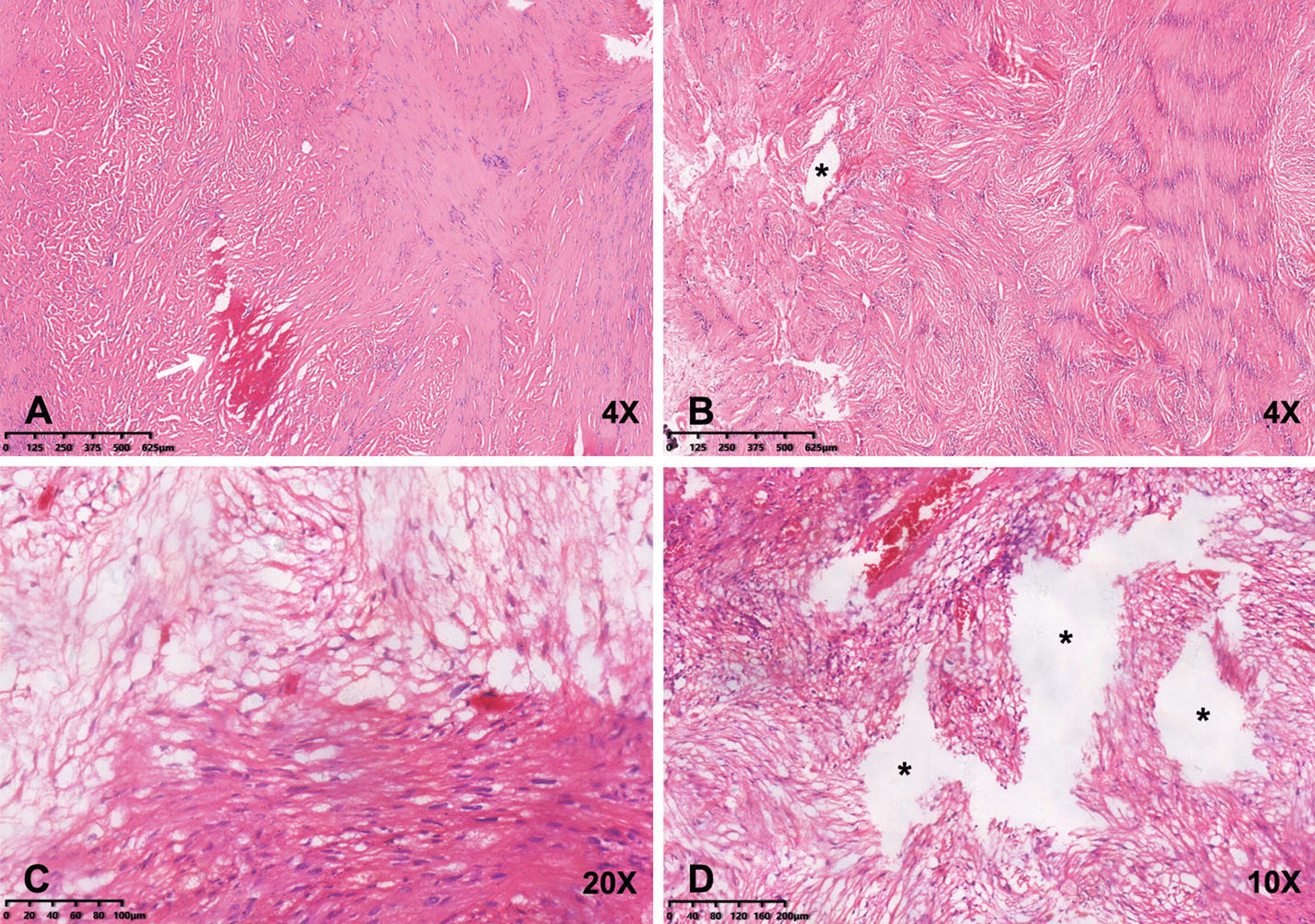
Photomicrographs of the histopathologic specimens demonstrate spindled cells arranged in short bundles and interlacing fascicles (cellular Antoni A areas), and the loose, myxoid matrix (Antoni B areas) (H&E) (**A**–**C**). Patchy hemorrhage (**A**, white arrow) and cystic degenerations (**B**, **D**, black asterisks) could be observed

Microscopically, the mostly part of neoplasms typically presented a biphasic histological pattern of high- and low-cellularity regions, with hemorrhage and cystic degeneration (Fig. 4). Moreover, the tumors indicated intense immunostaining for S100 and a relatively low Ki67 index (approximately 1–2%), implying that the tumors were neurogenic and the proliferation activity was relatively low.

## Discussion

Intra-parenchymal and intra-ventricular schwannoma is benign in nature, which is commonly reported in young adults without gender predilection[6]. Owing to its divergent location, size and texture, the clinical symptoms could be multiplied. For example, it could be asymptomatic when the mass effect was not obvious. Meanwhile, it could also present diverse neurological symptoms, such as dizziness, headache or vomiting, if one or more brain structures were oppressed or involved[7, 8].

The origin of intra-parenchymal and intra-ventricular schwannoma was still controversial due to the fact that no schwannoma cells could be found histologically in the central nervous system. As reported in previous studies, intra-parenchymal and intra-ventricular schwannoma could be divided into two subtypes, namely developmental subtype and non-developmental subtype. In the developmental subtype, the researchers assumed that the Schwann cells’ migration into the brain parenchyma during embryogenesis and the proliferating leptomeningeal cells’ transformation into Schwann cells may explain its origin. By contrast, in the non-developmental subtype, it is presumed that Schwann cells are formed by the differentiation of pluripotent neural stromal cells in the brain parenchyma or stemming from nerve plexus surrounding the subarachnoid vessels[9–13].

Due to the rarity and the limited reported cases, the radiologic features of intra-parenchymal and intra-ventricular schwannoma were investigated insufficiently. As observed in this study, the intra-parenchymal and intra-ventricular schwannoma commonly appeared as a solitary, rounded or oval, well-defined heterogeneous mass with calcification, cystic degeneration and delayed enhancement, It was consistent with previous studies[6, 14, 15]. In the present study, the intra-parenchymal and intra-ventricular schwannoma was solitary, oval and well-defined, which was potentially related to the incidence of a tumor capsule sign [4]. The common CT/MRI finding of inhomogeneity was presumably secondary to inadequate blood supply to the tumor center [16]. Calcification is another interesting feature of intra-parenchymal and intra-ventricular schwannoma. In the present study, calcification could be located in two cases, which was discrete or nodular, along the edge or in the center of the mass. Pathologically, calcification was concentric and laminated in shape. It was characterized by a Rosenthal fiber-rich reactive piloid gliosis, indicating chronicity in course of this disease[3].In addition, some uncommon imaging findings, such as hemorrhage, lobulation and fluid–fluid level, could also be obtained in some cases in this study.

Cystic degeneration with internal septa is another important CT/MRI characteristic of intra-parenchymal and intra-ventricular schwannoma. More interestingly, the cyst walls of these tumors are smooth, and the cystic areas are regular. As observed in this study, the majority of cases (5/7) were cystic and solid tumors, which correlated well with histopathological findings. Histologically, the schwannomas were composed of two main microscopic components, namely Antoni A areas (a highly cellular component) and Antoni B areas (a myxoid component). Hence, we speculated that the solid parts of the masses were suggestive of the highly cellular alterations (Antoni type A areas), while the cystic parts of masses may be corresponding to the myxoid components (Antoni type B areas)[3, 13]. Likewise, apart from the cystic change within the tumor, we could also obtain peritumoral cystic alterations. Presumably, the cystic regions surrounding the tumor were potentially related to an obstruction of CSF flow, which was secondary to the peritumoral adhesions[17].

In the present study, the intra-parenchymal and intra-ventricular schwannoma illustrated a characteristic enhancement pattern. It is evidenced by moderate to marked heterogeneous enhancement on the axial MR scan, and delayed enhancement on the coronal and sagittal MR images. The delayed heterogeneous enhancement of schwannoma was well documented in previous studies[5, 18].Zhang et al. ascribed the heterogeneous enhancement to a variation in the degree of cellularity and degenerative alterations[18]. As for the possible reasons for the delayed enhancement pattern, we considered that the mucous components of the tumors, which could result in a progressive infiltration and delayed washout of contrast agents, may be the substrates underlying this characteristic enhancement pattern[19, 20].

Intra-parenchymal and intra-ventricular schwannoma should be differentiated from intracranial solitary and cystic tumors. To be specific, the differential diagnosis of intra-parenchymal schwannoma should include ganglioglioma, pilocytic astrocytoma (PA), pleomorphic xanthoastrocytoma (PXA), cystic meningioma and dysembryoplastic neuroepithelial tumor (DNET) (Additional file 1: Table 1). Our findings that uncommon calcification and moderate to marked delayed enhancement could contribute to differentiating intra-parenchymal schwannoma from ganglioglioma, which is featured with temporal lobes, a high incidence of calcification, local thickening of peritumor cortex and mild heterogeneous enhancement[21, 22]. Septa, calcification and hemorrhage within the tumor or peritumoral edema are essential imaging features of intra-parenchymal schwannoma. Conversely, these findings could be hardly observed in PA[23, 24]. PXA usually occurs in the superficial parts of brain as well as temporal lobes, with mural nodules commonly observed and leptomeningeal involvement[25, 26]. It could be distinguished from intra-parenchymal schwannoma. Unlike intra-parenchymal schwannoma, cystic meningioma is featured by meningeal tail sign and bone hyperplasia adjacent to the skull. Furthermore, the signal or density of the solid component, equal to the adjacent gray matter, is also characteristic to this entity[26–28].DNET, mainly in children, is commonly occurs in frontal and parietal cortex with a typical triangle sign[29, 30]. However, these imaging features are rare in intra-parenchymal schwannoma. As for intra-ventricular schwannoma, it should be distinguished from ependymoma and choroid plexus papilloma (Additional file 1: Table 1). Irregular cystic degeneration, gravel-like calcification, signal heterogeneity, hemorrhage signal and plastic growth are essential imaging features for the diagnosis of ependymoma[31–34].They are the important differential points to distinguish ependymoma from intra-ventricular schwannoma. Choroid plexus papilloma (CPP) is characterized by a slightly high density on unenhanced CT images, a granular or mulberry-like mixed signal, intralesional flow voids on T2WI images and marked enhancement on contrast scans[33–35]. These are the unique points in the differential diagnosis of intra-ventricular schwannoma with CPP. What’s more, it is of great clinical significance to distinguish between benign and malignant Intracranial schwannoma. Intracranial malignant schwannoma usually presents with a large mass with irregular margins, mass effect, frequently accompanied by cystic degeneration, necrosis, calcification, and bleeding, and obviously uneven strengthening on enhancement scan[36].

However, this study has several limitations, as bellows: the population in this research was not large enough; the advanced functional imaging data (such as Magnetic resonance spectroscopy, Perfusion weighted imaging sequences and computed tomography perfuion) on the reported cases in our study were not available; the observed delayed progressive enhancement was just a kind of tendency or phenomenon, which should be further supported by the evidence from dynamic contrast-enhancement or perfusion weighted imaging.

## Conclusion

Intra-parenchymal and intra-ventricular schwannoma are rare and difficult to diagnose. It commonly appears as a well-defined unilateral mass with cystic degeneration, septa, and a characteristic, moderate to marked delayed heterogenous contrast-enhanced pattern on enhanced imaging.

## Supplementary Information

**Additional file 1.** Typical imaging findings of cystic tumours in brain.

## Data Availability

The original contributions presented in this study are basically included in the article/Supplementary Material; further inquiries could be directed to the corresponding author.

## Abbreviations

CT: Computed tomography
MRI: Magnetic resonance imaging
T2WI: T2-weighted images
T2-FLAIR: T2 fluid attenuated inversion recovery
PA: Pilocytic astrocytoma;
PXA: Pleomorphic xanthoastrocytoma
DNET: Dysembryoplastic neuroepithelial tumor
CPP: Choroid plexus papilloma
