# Supplementary material for: CT and MRI findings of intra-parenchymal and intra-ventricular schwannoma: a series of seven cases
Source: BMC Med Imaging. 2022 Nov 17;22:197. doi: 10.1186/s12880-022-00917-z (PMC9670463; doi:10.1186/s12880-022-00917-z)
Supplement: Supplementary file 1 — Additional file 1. Typical imaging findings of cystic tumours in brain. [file 12880_2022_917_MOESM1_ESM.docx]

| **Additional file 1: Table 1. Typical imaging findings of cystic tumors in brain.** | |
| --- | --- |
| **Entities** | **Typical imaging findings** |
| Schwannoma | Well-defined margin, smooth cyst wall, septa, delayed moderate to marked enhancement |
| **Intra-parenchymal cystic tumors** | |
| Ganglioglioma | Temporal lobes mostly involved, a high incidence of calcification, local thickening of peritumor cortex, mild heterogeneous enhancement |
| Pilocytic astrocytoma | Children and adolescents mostly affected, infrequent calcification, septa, hemorrhage and peritumoral edema |
| Pleomorphic xanthoastrocytoma | Superficial parts of brain as well as temporal lobe mostly affected, featured by mural nodules and leptomeningeal involvement |
| Cystic meningioma | Meningeal tail sign, the signal density of tumor solid component being equal to the adjacent gray matter, bone hyperplasia adjacent to the skull |
| Dysembryoplastic neuroepithelial tumor | Mainly in children, frontal and parietal cortex mostly involved, featured by a typical triangle sign |
| **Intra-ventricular cystic tumors** | |
| Ependymoma | Irregular cystic degeneration, gravel-like calcifications, hemorrhage, plastic growth |
| Choroid plexus papilloma | Slightly high density on CT images, a granular or mulberry-like mixed signal on T2WI images, marked enhancement |
